# Measuring What Outcomes Matters Most to People When Accessing Suicide Postvention Support: A Qualitative Study

**DOI:** 10.1007/s10597-025-01452-1

**Published:** 2025-01-21

**Authors:** Bess Jackson, Sarah Wayland, Shelley-Anne Ball, Myfanwy Maple

**Affiliations:** 1 University of New England, School of Health, Armidale, NSW Australia; 2 StandBy Support After Suicide, Maroochydore, QLD Australia; 3 Central Queensland University, School of Nursing, Midwifery and Social Sciences, Rockhampton, QLD Australia

**Keywords:** Suicide postvention, Support, Outcomes, Outcome measures, Lived experience

## Abstract

Funding for suicide postvention services, which provide support after a suicide death, has increased in Australia and globally. This rise accompanies a need to demonstrate outcomes of support. However, articulating and quantifying these outcomes presents ethical and logistical challenges. Funders' priorities may differ from those of service users. To discern the value and explore effective measurement of postvention outcomes, focus groups were conducted with postvention staff and lived experience representatives from an Australian postvention service. Transcripts were analysed using Braun and Clarke’s Reflexive Thematic Analysis. Results highlighted the complex context of measuring outcomes in suicide postvention and emphasized the need for flexible approaches to service provision and outcome measurement. The study suggests that the most significant benefits, as perceived by participants, are the ‘flow-on’ effects of postvention. It supports the notion that outcome measures require careful consideration, with trade-offs evaluated to understand what is truly valuable in suicide postvention services.

**Supplementary Information:**

The online version contains supplementary material available at 10.1007/s10597-025-01452-1.

## Introduction

In recent decades, the understanding of suicide and its far-reaching impacts has grown due to heightened research, both within Australia and on a global scale (Maple et al., 2018). In 2022, over 3,200 lives were lost to suicide in Australia (Australian Bureau of Statistics, 2022). Current approximations propose that for each suicide, as many as 135 individuals may have been acquainted or close with the deceased (Cerel et al., 2019) and will experience varying levels of impact and/or bereavement (Cerel et al., 2014).

The impacts of suicide bereavement are well documented throughout the literature and in lived experience accounts. People bereaved by suicide are at higher risk of suicide themselves (Maple et al., 2017). When compared with other forms of bereavement, the experience of rejection, guilt, stigmatisation, and responsibility may be more common among the suicide bereaved (Kõlves et al., 2020), potentially contributing to the higher instances of complicated grief (de Groot & Kollen, 2013). It is vital that the support received in the wake of a suicide loss, known as suicide postvention, is sensitive to the nuances of suicide bereavement and free of stigmatising judgement (Kaspersen et al., 2022).

In order to promote the provision of appropriate, timely support to people who are bereaved by suicide, postvention support is increasingly included as an integral element of national suicide prevention plans (Department of Health, 2020; Ministry of Health, 2019). Australia has witnessed a significant investment in suicide postvention, with $14.4 million invested to support the continuation of postvention services nationally over 2023–2025 (Suicide Prevention Australia, 2023).

To support the evidence-based allocation of resources, it is common for funders to seek demonstration of the outcomes achieved by a publicly funded service (Adams et al., 2015). The outcomes may be at an individual-, program-, or population-level and captured using outcome measurements (Seivwright et al., 2016). The term 'outcome measurements' encompasses all forms of tools that measure the impact a service makes in the lives of the people it supports (Adams et al., 2015). The practice of measuring outcomes and meaningfully using their insights to inform care is not yet embedded within the Australian community sector (Seivwright et al., 2016). Within the field of suicide postvention, the most appropriate approach to understanding and measuring the outcomes of those who access services remains unclear and requires consultation of the literature and engagement with those with a lived experience of suicide bereavement (Pitman et al., 2016).

A scoping review conducted by Jackson et al. (2024) identified ways in which outcomes and experiences are captured in a range of interventions supporting people after a sudden, traumatic loss. The approaches were inconsistent across the programs, and often involved a battery of outcome measures tools designed to detect reductions in negative affects or experiences. Very few papers appeared to seek service user input into the design of the outcome measurement approach, despite the benefits of including lived experience perspective in all elements of program design (Brett et al., 2014).

In the design and development of outcome measurements, understanding the views of all stakeholders may lead to better implementation and, by extension, the generation of more meaningful and reliable data (Wolpert, 2014). Crawford et al. (2011) undertook a consultation process whereby consumers of mental health services were asked to rate the acceptability and relevance of two dozen outcome measure tools. Participants expressed concern about the ability of certain outcome measures to comprehensively capture their experiences. Specific to suicide postvention, a similar approach was adopted by Pitman et al. (2016) in which lived experience representatives were consulted about the design of a postvention service and acceptable outcome measure tools. Generally, the group acknowledged the necessity of outcome measures and identified five key outcome measures which they felt postvention may support (isolation, stigma, psychological health, day-to-day social functioning, and functioning in a work or caregiver role); the group did not, however, discuss how these outcomes are best measured or assessed. The current study sought to build on previous research by holding focus groups aimed at exploring preferred approaches to measuring outcomes of suicide postvention support.

## Methodology

Focus group methodology was selected as it provides an opportunity for participants to discuss their perspectives as well as providing a platform for interaction (Davidson et al., 2022). Focus groups are particularly valuable when researching a stigmatising experience, such as suicide bereavement, as participants may feel validated by the views and opinions of others (Ussher et al., 2020).

The data generated through the focus groups was analysed following Braun and Clarke’s (2006, 2019) reflexive thematic analysis (TA) methodology. Broadly speaking, TA encompasses a family of methods used for identifying, analysing, and reporting patterns of meaning (themes) in qualitative data (Braun & Clarke, 2014). Reflexive TA involves six phases of analysis that, although presented linearly, are moved between in a recursive manner (Braun & Clarke, 2006). We found ourselves oscillating between phases or, quite often, sitting in multiple phases simultaneously. By moving through the phases, we constructed themes from the information shared by the participants, comprised of threads of patterned data that were woven together, bound by a shared centralised concept. The themes contribute to the answering of our core research questions:

- What are the essential elements in postvention support?
- What approach should be taken to measure the outcomes associated with postvention support?
- What are the barriers or issues associated with this?

### Positionality

The conceptualisation of reflexive TA as a method, as opposed to a fully embedded methodology, allows flexibility in the theoretical lens from which the analysis is approached (Braun & Clarke, 2021). Braun and Clarke (2021) have discussed several theoretical assumptions that must be established when conducting reflexive TA. In alignment with the qualitative paradigm integral to reflexive TA, we adopted a constructionist epistemology, recognising the bidirectional relationship of language and experience (Byrne, 2022). As such, when coding the information produced in the focus groups, the recurrence of the information was considered, however, greater weight was placed on the meaning and meaningfulness of the coded information by considering the tone, emphasis, non-verbal cues, and (dis)agreements displayed by the participants. We adopted an experiential orientation to data interpretation, understanding that the information furnished by the participants reflected their subjective personal states (Braun & Clarke, 2014).

Identifying one’s worldview and experiences provides a framework to the research and helps situate it within the broader context (DeCuir-Gunby & Schutz, 2017). Our academic grounding includes social work and psychological science, with our collective professional backgrounds encompassing clinical work, academia and research, service design and commissioning, and program management. We approached the research informed by lived experiences of suicide, but not leading with lived experience.

### Focus Group Methodology

The original study was designed around three distinct groups, all of which related to the same Australian postvention service: staff delivering suicide postvention; people currently receiving postvention support; and a lived experience advisory group (LEAG). These groups were selected as they are the most impacted by the research outcomes, a hold potentially differing viewpoints towards outcome measurements in postvention. Various recruitment strategies were implemented, tailored to each group, aiming to recruit 10–12 participants in each group. The LEAG and staff cohorts received the invitation to participate in the focus groups via email following a presentation at their respective regular meetings. In accordance with our ethics approval, the recruitment strategy for service users relied on staff inviting individuals they were currently or had previously supported, ensuring their bereavement had occurred more than 12 months prior. When no interest was received for the focus groups, we offered individual interviews in the hopes this may elicit interest. Unfortunately, despite significant efforts to incorporate the perspectives of people currently receiving postvention support, their direct input was unable to be secured and therefore was not able to be included in the study. The impacts and potential limitations are discussed below.

The first author (BJ) developed an interview guide according to the framework proposed by Davidson et al. (2022) which we reviewed through an iterative process (Online Resource 1). The focus groups were held over video conferencing software Microsoft Teams and were automatically recorded and transcribed. Participants were encouraged to respond to the questions verbally or by using the chat function. Additionally, participants were invited to provide further thoughts via email following the focus groups, however none chose to do so. The groups were moderated by the first author (BJ), with the second author (SW) assuming note-taker role for one of the groups. Due to unforeseen circumstances, a note-taker was not available for one group, leading to the moderator (BJ) assuming both roles and using the recording to augment the field notes. We intentionally incorporated reflexivity rounds between each focus group and kept detailed notes throughout.

The video recordings of the focus groups were re-watched, and the transcripts and chat logs were reviewed, amended, and annotated, thus beginning the first phase of analysis, familiarisation with the data (Braun & Clarke, 2006). Field notes were similarly included in the analysis, and entries in our reflexivity journal were used as a mirror throughout to reflect on our role as researchers in the inquiry process.

### Analysis

The transcripts were uploaded into NVivo, and data familiarisation continued through the re-reading and annotating of the transcripts. Codes were generated by the first author (BJ) by highlighting sections of the text deemed relevant to the research questions and attaching a clear label. The analysis was conducted using a predominately inductive approach, with no attempt to fit the codes into an existing theory. However, as per Byrne’s (2022) approach, a degree of deductive analysis was used to ensure that the codes being generated were contributing to the research questions. The codes were both semantic, focused on the explicit meaning of the participants’ words, and latent, related to the underlying meaning of the spoken words. Candidate themes were developed, which provided a framework for codes to be grouped, enabling the identification of shared meanings across the extracted coded data. In an iterative process with the wider research team, the codes were re-worked; similar codes were consolidated, ambiguous codes were reworded, and codes lacking meaningful contribution were removed. Following the approach employed by Trainor and Bundon (2021), the coded transcripts were juxtaposed against clean copies. Subsequently, the clean copies were re-coded using the revised codes, while also considering the original coding. The candidate themes and subthemes were revised and restructured, leading to the naming and defining of the final themes.

## Results

A total of six postvention workers and seven LEAG members expressed interest in participating in their respective focus group, however one person from each group was unable to attend on the day. The final participants included two males and three females in the postvention worker focus group, and three males and three females in the LEAG group.

**Fig. 1:**
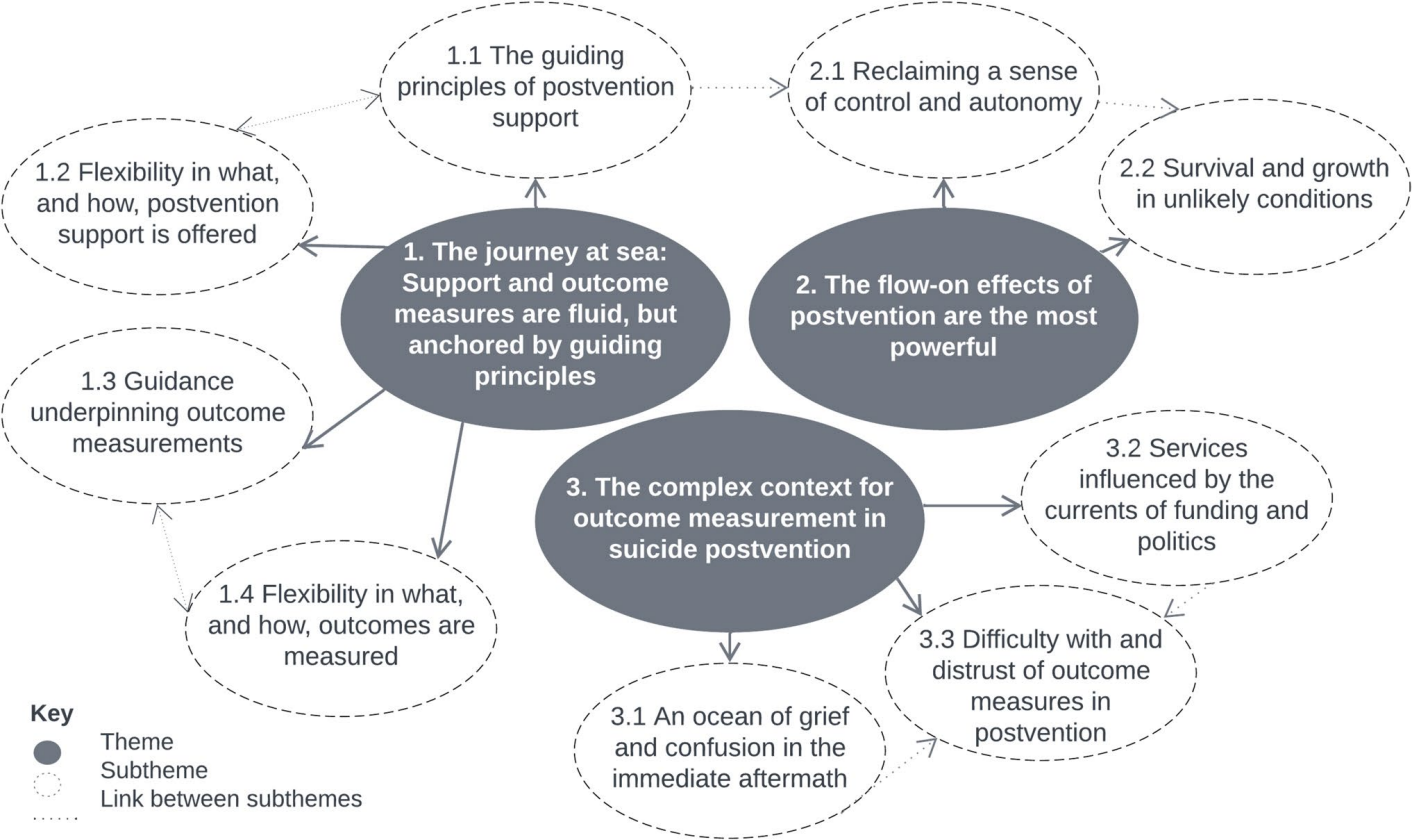
Thematic map of themes and subthemes; created using Lucid (lucid.co)

The finalised themes are structured such that they describe the essential elements of postvention support and the measurement of support outcomes (theme 1), discuss the perceived outcomes of such support (theme 2), and describe the context of outcome measurement in suicide postvention (theme 3). Each theme has subthemes that further delineate the nuances of the overarching concept, which are illustrated with pseudonymised participant quotes below. Refer to Fig. 1 for a graphical depiction of the relationship between the themes and subthemes.

## The Journey at Sea: Support and Outcome Measures are Fluid but Anchored by Guiding Principles

A sense of navigating a metaphoric ‘journey lost at sea’ after a suicide bereavement forms the basis of this theme to describe postvention support and the subsequent measuring of support outcomes. This theme contributes to research questions (a) and (b) by capturing the guiding principles, as shared by focus group participants, that are essential to supporting people after a suicide loss.

### The Guiding Principles of Postvention Support

This subtheme ties together the principles that underpin postvention service provision, centred around the idea that, above all else, the individual should be at the centre of their care. The person should be listened to, and gentle guidance offered through a lens of lived experience, with emphasis on the continuity of connection between worker and individual. Anecdata shared by the participants suggested that individuals stay engaged with supports for longer when they remain connected with the same worker.

The value of having somebody available to listen was strongly emphasised by the participants. They shared that a listening and empathetic ear was essential after experiencing a suicide loss, and that often family and friends were unable or unwilling to provide this. Similarly, the importance of round-the-clock support was noted, acknowledging that the profound grief experienced by individuals can manifest at any hour and significantly impair functioning. The importance of having the right person delivering postvention was consistently emphasised by the participants. The value of being supported by somebody who understands the complexity of suicide bereavement cannot be overstated, whether that be through lived experience or professional, empathetic expertise.

“*A large…. thing is just someone to listen, someone who understands.*”

—Olivia, LEAG member

Another essential element in postvention support is that the person is positioned as the expert of their experience. Support that is perceived as dictating or prescriptive is unhelpful; rather, guidance formed on the basis of genuine listening is preferred.

“*The person is, kind of, the expert in their experience; … we are not there to push or pull or fix or force.”*

—Ava, postvention staff

### Flexibility in What, and How, Postvention Support is Offered

Building on the notion that the person is the expert in their experience, this subtheme speaks to the flexibility that is required in the way that support is offered. There were distinctions drawn between the support needs of different groups, with men, LGBTIQA + communities, and people of culturally and linguistically diverse backgrounds identified as groups with specialised support needs.

As well as variance across groups, variance between individuals similarly requires a nuanced approach to support. An individual's personality and preferences will inevitably shape the kind of support they are willing to accept and find beneficial. Moreover, their unique circumstances will significantly influence their support requirements. Given the ripple effect of a suicide loss, many people may find themselves in need of postvention assistance and support.

“*We're supporting witnesses as well as people bereaved, so their needs are a little bit different.”*

—Thomas, postvention staff

The support needs of individuals can similarly differ. The intensity and frequency of support services tend to fluctuate, with individuals disengaging from support at various times. A marked drop off was noted after one year of support. However, this pattern is not universal, as some individuals benefit from sustained engagement while others prefer to avoid reflection or reminders. There was recognition of the importance for support to remain available over an extended period, possibly beyond what would be expected, to accommodate varying preferences and needs for ongoing assistance.

To cater to the diverse needs of individuals, participants spoke of the balance between asking individuals about their support needs and offering options and suggestions. The act of asking enables participants to identify their priorities and empowers them to take ownership of their journey. Conversely, there may be instances where specific support options are recommended, as many may find themselves overwhelmed and lacking clarity on their own needs.

*“It is awesome to be a listener. It's awesome to ask people what they want and need, but most of the time they're going to turn around and say “*well, I don't know what I want and need” *and so it's great to have some options at our disposal.”*

—Mason, LEAG member

### Guidance Underpinning Outcome Measurements

Undercurrents of apprehension regarding the introduction of outcome measures were discerned, often related to concerns that funders’ requirements would be prioritised over service users’ needs, prompting participants to share guidance aimed at ensuring the safety and appropriateness of outcome measurement practices. Participants emphasised the importance of both the outcomes themselves and the measurement tools employed aligning with the theoretical framework and values of the program. Alignment with program theory is important, as the theory outlines the rationale behind anticipated changes, with outcome measures serving as a metric of such changes. Alignment with the program's values is of equal significance, as it ensures that the outcome measure approach matches other aspects of the program.

*“We really just need to align with those values of being very client centred and I think the danger in* “OK, now you need to give us some information because **we** need it” *… being too formal too early, I think would jeopardise that …. beautiful kind of feel that we have with the program.”*

—Mia, postvention staff

As illustrated in the quote, there was a belief shared by participants that outcome measures must be of reciprocal benefit to the person and the program. They advocated for an approach that remains person-focused and offers benefits to the person. Discussion centred around the incorporation of outcome measures into the support in a manner that appears informal and unstructured, yet effectively captures the individual's outcomes. Over the course of the discussions, participants acknowledged that outcome measures could be integrated into care, rather than being perceived as an addendum. An emphasis was placed on the principle that outcome measures should have minimal impact on both clients and staff, as well as on the therapeutic relationship between them.

### Flexibility in What, and How, Outcomes are Measured

This subtheme was constructed around additional insights shared by participants, which they perceived as mitigating their concerns regarding outcome measurements. These insights were grouped together under this subtheme to underscore the necessity for flexible and adaptable approaches to outcome measurements. Both focus groups reached a consensus that given the considerable variability in support and support needs, customisable outcome measures would be helpful. There was discussion around the benefits of empowering individuals to define their own intended outcomes, and then crafting a measure around this. Although there was an oblique acknowledgment that ‘tangible data’ is needed for reporting purposes, there was a lack of agreement regarding the benefits of standardised measures for facilitating comparability both within individuals and across different programs.

Emphasis was placed on the need for a sensitive approach to outcome measures, recognising the complex nature of suicide bereavement and grief. An alternative proposal that emerged during discussions involved the possibility of staff members completing some form of subjective measure, rather than passing it on to the participant.

*“Obviously, you're not gonna slap a survey down in front of someone who's just been bereaved … But the support workers on the ground, I would like to think most of these people doing that work are able to criticise themselves and would want to do it better … And if you've got the same support worker dealing with one person, say, for example, over a year … they're gonna see what progress has made has been made with that person.”*

—George, LEAG member

The necessity for flexibility in the timing of outcome measures, with due consideration for the individual's grieving process, was a central idea shared throughout the discussions. Participants expressed the importance of ensuring that outcome measures remain optional and are not mandated as a prerequisite for accessing support. Suggestions were made to both stagger the administration of outcome measures away from the provision of support and also to integrate them within the support. Regardless of the timing, respect for the individual’s grief and sensitivity to their capacity to engage in outcome measurements are paramount considerations.

*“[We must be] really mindful of their capacity at different levels and at different stages of the grief. What can they actually engage with at that time? And that may even be different for different people.”*

—Mia, postvention staff

Recognition was given to the need for flexible modalities of outcome measures, tailored to the preferences of the individual. Given the potentially limited size of the postvention workforce, careful consideration is warranted regarding the individual's comfort level in disclosing honest outcomes to someone who has been closely supporting them. Proposed modalities included written assessments, face-to-face verbal discussions, phone-based verbal discussions, or visual aids, offering a range of options to suit varying communication preferences.

## The Flow-On Effects of Postvention are the Most Powerful

As discussed in subtheme 1.2, postvention support can be conceptualised not as the rescue of someone at sea, but as the giver of tools to guide a person on their journey. This sentiment is articulated elegantly by Putri et al., (2024, p. 2): “The unique and complex emotional landscape that suicide loss survivors traverse demands an approach that empowers them to navigate their grief journey with agency and resilience”. The current theme further contributes to research question (b) by building on the approaches to outcome measurement discussed in 1.3 and 1.4 and describing how the indirect or flow-on outcomes have the greatest impact on service users.

### Reclaiming a Sense of Control and Autonomy

This subtheme centres around one of the most powerful outcomes of postvention—a regained sense of control or autonomy. The participants alluded that while the direct impact of postvention support may be difficult to identify, the flow-on outcomes related to regained autonomy are significant. This was tied with the dissemination or facilitation of knowledge, and the confidence and sense of control that this may bring with it.

*“[The sharing of knowledge] really seems to resource some people, so that they are quite capable of moving into some degree of coping …. I think of it almost as somebody throwing a …. rescue tube their way and they can kind of go ‘*oh now I can relax, I can probably swim out of this in a minute’.*”*

—Noah, LEAG member

A sense of control can also stem from knowing that assistance is accessible; often, it is the perception of support that is enough. Regardless of whether support is accessed, simply knowing it is available provides reassurance and sense of power for some individuals.

The encouragement of self-care is another aspect that highlights the indirect yet substantial flow-on effects of postvention support. Postvention serves as a catalyst for promoting and reinforcing good self-care practices, either by serving as a reminder or by modelling such behaviours. Self-care may include the identification of coping strategies, which are crucial during the overwhelming initial stages of bereavement. It was noted that while most individuals are aware of the concept of self-care, it often gets deprioritised during challenging times, making postvention a valuable reminder of its importance.

*“Self-care impacts on everything …. If you've got good self-care practices, it just makes everything else so much easier. And I think in terms of that being attributed to support, I do think that we are instrumental in that process.”*

—Mia, postvention staff

In any support program, identifying the specific changes attributed solely to the support provided, while excluding other external factors, poses a considerable challenge. In postvention, given its significant indirect or flow-on effects, this task becomes even more difficult. Participants expressed this sentiment, highlighting the complexity of discerning which outcomes can be directly attributed to postvention and which outcomes postvention contributed to indirectly. The natural grieving process was mentioned by participants, prompting discussions about the role of postvention in shaping or impacting that process.

*“I think the thing about grief is that people generally will find their own way through anyway. Some people don't, and the grief does become complicated. But I think [postvention support] …. can and does play a really important role in helping that process; just by giving the information and normalising things, it helps people along in that process.”*

—Thomas, postvention staff

### Survival and Growth in Unlikely Conditions

Building on the central idea of subtheme 2.1 that postvention contributes to a sense of reclaimed autonomy, this subtheme captures the notion that postvention creates the foundations and opportunities for growth. Post-traumatic growth is often characterised by newfound meaning, direction, or appreciation for life after experiencing a trauma. Postvention support doesn’t necessarily force a person in this direction but rather provides the fertile ground upon which growth can potentially occur.

“*By the time I found [postvention service] … they were able to support me to be able to work out what I'm going to do in my life, because my whole life was shattered.”*

—Sophia, LEAG member

Another way in which postvention supports growth is through facilitating connections with others with a similar experience, which can further the healing and meaning-making processes. The peer workforce, which often makes up a portion of postvention staff, can offer guidance and reassurance, serving as role models who demonstrate that it is possible to survive the initial stages of grief and emerge resilient.

*“[Lived experience workers are] vital to show people are they can actually survive something that is almost impossible to survive.”*

—George, LEAG member

Another growth outcome associated with postvention is the growth of the future workforce. Engagement with postvention workers has the potential to inspire individuals who have recently experienced suicide bereavement to pursue roles as postvention workers themselves.

*“I just find that an interesting … unintended element of the [postvention] support which is that you actually create our future workforce.”*

—Ava, postvention staff

## The Complex Context for Outcome Measurement in Suicide Postvention

The environment in which the suicide postvention sector operates is complex, and this theme addresses the challenges and sensitivities surrounding the implementation of outcome measures, providing context to answer research question (c).

### An Ocean of Grief and Confusion in the Immediate Aftermath

Illustrating the complex context in which suicide postvention support is delivered, this sub-theme describes the grief and confusion and “busyness” of a traumatic loss. The participants spoke to the perplexing and painful aftermath of losing a person to suicide, and how this often makes navigating services difficult. They noted the challenges associated with an increased police presence, navigation of the coroner's process, liaison with financial institutions and superannuation, feelings of responsibility and guilt, the experience of stigma, and financial stresses tied to the loss.

*“It's expensive to lose somebody to suicide and there's not that many support options.”*

—Ava, postvention staff.

### Services Influenced by the Currents of Funding and Politics

Subtheme 3.2 is constructed around the participants' discussions of government influence and funding, and the limitations this poses on postvention services. It emphasises how the political and funding environment of postvention complicates service delivery, making outcome measures more complex and challenging to implement. The dialogue centred around the notion that the funders of postvention services have their own needs, and that this can limit the autonomy and ownership of services. There appeared to be a conflation between government-set outcome expectations for programs and the outcome measures passed on to participants.

*“Funding often needs to be tied to the empirical measures, so some objective value is needed to decide whether support is meeting KPIs.”*

—Noah, LEAG member.

### Difficulty with and Distrust of Outcome Measures in Postvention

This subtheme is organised around the inherent difficulties of measuring or capturing outcomes in suicide postvention, and the undercurrents of discomfort or fear that relate to it. Concern that government-funder requirements would impact outcome measures was apparent. Relating to the central concept of subtheme 1.1, participants expressed concern about posing outcome-related questions in times of heightened vulnerability.

*“I think [the time] our support is the most essential is also the time at which it would probably be the least appropriate to ask these sorts of questions.”*

—Ava, postvention staff

The participants discussed the concept of ‘reasonable expectations’ within the context of a postvention program. Specifically, they questioned what changes or outcomes could reasonably be expected from a service supporting individuals after a suicide loss. A subtle unease was detected, with participants expressing concern that should the measures fail to demonstrate improvement, service provision or resource allocation may be impacted. It was suggested that services may be intentionally or unintentionally directed toward those who exhibit ‘better’ outcomes. Participants noted that a person’s support needs may fluctuate, which is related to the non-linearity of grief, rather than serving as a measure of success or failure of a postvention service.

*“It is completely reasonable that someone who is given good support collapses and has a terrible period of time because they're free to actually, you know, express their grief in an individual way…. That's not a failure [of service], right?”*

—Noah, LEAG member

## Discussion

Reflexive TA acknowledges the researcher as having an active role in the interpretation of meaning, leading to findings that “inevitably and inescapably bear[] the mark of the researcher(s)” (Braun & Clarke, 2014, p. 1949). As such, it is vital that we acknowledge our positionality and the assumptions we have made in relation to the research question (Braun & Clarke, 2021). The research sought to answer how outcome measures could be embedded in postvention service delivery, rather than *if* they should. Historically, outcome measures have been integrated into Australian mental health public policy without much critical reflection on the methodology or rationale behind their implementation (Oster et al., 2023). There is a perception among community mental health stakeholders that outcome measures primarily serve to gather data to satisfy funders and fulfil reporting requirements (Queensland Alliance for Mental Health [QAMH], 2019). Some participants of the focus groups shared a similar sentiment. In the suicide postvention space, concerns have been raised regarding the framing of certain outcomes, which may inadvertently steer the service toward a clinical approach or place undue emphasis on deficits (Weier et al., 2022). Notwithstanding, leading suicide postvention researchers have called for a core set of outcome measurements to be introduced in the suicide bereavement context (Andriessen et al., 2019a, 2019b). Partly in response to these calls, the research forms part of a PhD project, the aim of which is to recommend to a national postvention service an evidence-based, suitable, and sensitive approach to outcome measurement. While the absence of challenges to the concept of outcome measures does not necessarily diminish the analysis, it is vital to acknowledge the perspective that was prioritised in the conversations.

The analysis of the focus group conversations underscores the complex nature of embedding outcome measures into suicide bereavement services. Measuring outcomes related to bereavement interventions is inherently difficult (Schut & Stroebe, 2011). An added layer of complexity in postvention is the involvement of government funders, who often seek demonstration of particular outcomes (Adams et al., 2015). As a result, their requirements can influence the selection of outcome measures, which may not fully consider the nuanced sensitivities required for individuals bereaved by suicide. Additionally, the request for demonstration of outcomes is underpinned by the assumption that the outcomes are attributable to the support provided; by extension, this assumes that a lack of positive outcomes is due to low quality and performance on behalf of services and their staff (Oster et al., 2023). These concerns potentially contributed to the subtle yet discernible apprehension, evident in the focus group discussions, that outcome measures could impact the funding of services or compromise the individualised nature of support provision.

The individualisation and person-centredness of support was emphasised by participants as the most essential element of postvention. Rather than a particular type or function of support, it was the adaptability to meet the person’s needs that was identified as most important. This aligns with conclusions drawn from a 2019 systematic review, which indicated that postvention programs offering a range of support tailored to participants' individual needs tended to demonstrate higher levels of effectiveness (Andriessen et al., 2019a, 2019b). Similarly, Abbate et al. (2022) found that the type of support was less important when considering the effectiveness of postvention services, but rather whether the support led to a sense of connection and belongingness. This lends support to the notion that the flow-on effects of postvention are the most powerful. However, in terms of measuring outcomes, this presents potential challenges, as the impacts of support may not be fully realised until after the individual has disengaged.

Like the essential elements of postvention, no singular approach to outcome measurement was universally preferred. Instead, there was consensus from both the staff and LEAG groups on the importance of tailoring outcomes to the individual and delivering them in a manner that aligns with their preferences. Various options such as written surveys, verbal assessments, and visual aids were discussed, with a focus on minimising the burden on both the individual and staff. Concerns related to the burden of outcome measures are not unique to suicide postvention; they have been documented in mental health (QAMH, 2019) and broader health contexts (Williams & Thompson, 2018). Rather than representing an insurmountable barrier, herein lies an opportunity to engage staff in an alternative narrative that highlights the opportunities in the implementation of routine outcome measures. The focus groups did identify some of these potential opportunities when discussing the incorporation of outcome measures as an integral component of the individual's care, rather than being seen as an addition to it.

A preference for unique or customisable outcome measures was shared by the focus group participants, underpinned by the assumption that standardised measures may fail to capture the entirety of individuals' experiences (Crawford et al., 2011). This concern was also identified by Greenhalgh et al.'s (2018), who found that some individuals found the wording of standardised health care measures harmful, while customised outcome measures were perceived as more valid and less distressing. Within the focus groups, there was an emphasis on empowering individuals to identify their personal definitions of outcomes, which has the potential to inform care and ongoing service development (Roe et al., 2022). Barrenger et al. (2019) advocate for a similar ‘bottom-up’ approach in mental health peer support, consulting service users on their prioritised outcomes and then implementing measures based on this. While this approach holds promise, it may lead to the selection of inconsistent outcome measure approaches, thereby limiting comparability across programs. Comparability is a priority within the postvention space, with leading researchers calling for the “development of a “core outcome set” for suicide bereavement interventions [which] could facilitate collection and reporting of comparable effectiveness data” (Andriessen et al., 2019a, 2019b, p. 13). Perhaps the antidote to this tension lies in Greenhalgh et al.’s (2018) conclusion that the trusting relationship between service users and staff may hold greater significance than the specific content of outcome measures.

## Limitations

The research has some potential limitations which must be noted, the most impactful of which is the absence of current service users. Describing outcome measures or measurement is conceptually challenging and perhaps not appealing to those who have recently used the service. Despite significant efforts to recruit current service users, none were able to be recruited. Lived experience voices were represented with the inclusion of the LEAG participants, some of whom had previously accessed the postvention service. Therefore, although a different lens may have been applied, there was some service user representation. Due to difficulties encountered in the recruitment process, the participant groups were smaller than intended. Initially, the aim was for focus groups consisting of 10–12 participants. However, recruitment for the LEAG was constrained by the existence of a pre-established group. There were challenges eliciting interest from staff, which may be indicative of the pervasive sense of busyness prevalent in the sector. All participants were associated with the same national postvention service; consequently, it is possible that their perspectives may align in a similar direction or are unique to the culture of the service. The use of videoconferencing potentially stifled group dynamics and limited the recognition of non-verbal cues. However, videoconferencing also enabled greater geographic representation and permitted recording and replaying, thereby mitigating the absence of the notetaker in one of the groups. Finally, although reflexive TA acknowledges the researcher as having an active role in the interpretation of the data, our use of a single coder may have led to potential bias in the interpretation. To minimise potential bias, the coding process was overseen by the wider research team and themes were generated at a team-level.

## Conclusion

The focus groups highlight the need for further research into the nuanced views of stakeholders regarding the tools used to measure outcomes in the context of suicide postvention. It is essential to recognise that different stakeholder groups may hold different views, influenced by their ontological and epistemological perspectives. Therefore, additional research aimed at building consensus among various stakeholder groups may provide the necessary insights into their shared priorities for outcome measurement in suicide postvention. The implementation of appropriate and acceptable outcome measures will contribute towards the suicide postvention evidence base, providing greater insights into what elements of this important support works for whom.

## Supplementary Information

Below is the link to the electronic supplementary material.

Supplementary file1 (PDF 24 KB)

## Data Availability

Due to the sensitive nature of the data and the potential for participant identification, the transcripts are not publicly accessible. However, the focus group interview guide can be found in Online Resource 1.

## References

[CR1] Abbate, L., Chopra, J., Poole, H., & Saini, P. (2022). Evaluating postvention services and the acceptability of models of postvention: A systematic review. *Journal of Death and Dying*. 10.1177/00302228221112723 35790465 10.1177/00302228221112723 PMC11487908

[CR2] Adams, S., Flatau, P., Zaretzky, K., McWilliam, D., & Smith, J. (2015). *Measuring the difference we make: the state-of-play of outcomes measurement in the community sector in Western Australia* [Report]. Centre for Social Impact. https://apo.org.au/node/57579

[CR3] Andriessen, K., Krysinska, K., Hill, N. T. M., Reifels, L., Robinson, J., Reavley, N., & Pirkis, J. (2019a). Effectiveness of interventions for people bereaved through suicide: A systematic review of controlled studies of grief, psychosocial and suicide-related outcomes. *BMC Psychiatry,**19*(1), 49–49. 10.1186/s12888-019-2020-z 30700267 10.1186/s12888-019-2020-z PMC6354344

[CR4] Andriessen, K., Krysinska, K., Kõlves, K., & Reavley, N. (2019b). Suicide postvention service models and guidelines 2014–2019: A systematic review. *Frontiers in Psychology,**10*, 22. 10.3389/fpsyg.2019.02677 31849779 10.3389/fpsyg.2019.02677 PMC6896901

[CR5] Australian Bureau of Statistics. (2022). Causes of Death, Australia. https://www.abs.gov.au/statistics/health/causes-death/causes-death-australia/2022.

[CR6] Barrenger, S. L., Stanhope, V., & Miller, E. (2019). Capturing the value of peer support: Measuring recovery-oriented services. *Journal of Public Mental Health,**18*(3), 180–187. 10.1108/JPMH-02-2019-0022

[CR7] Braun, V., & Clarke, V. (2006). Using thematic analysis in psychology. *Qualitative Research in Psychology,**3*(2), 77–101. 10.1191/1478088706qp063oa

[CR8] Braun, V., & Clarke, V. (2014). Thematic analysis. In T. Teo (Ed.), *Encyclopedia of critical psychology* (pp. 1947–1952). Springer.

[CR9] Braun, V., & Clarke, V. (2019). Reflecting on reflexive thematic analysis. *Qualitative Research in Sport, Exercise and Health,**11*(4), 589–597. 10.1080/2159676X.2019.1628806

[CR10] Braun, V., & Clarke, V. (2021). One size fits all? What counts as quality practice in (reflexive) thematic analysis? *Qualitative Research in Psychology,**18*(3), 328–352. 10.1080/14780887.2020.1769238

[CR11] Brett, J., Staniszewska, S., Mockford, C., Herron-Marx, S., Hughes, J., Tysall, C., & Suleman, R. (2014). A systematic review of the impact of patient and public involvement on service users, researchers and communities. *Patient,**7*(4), 387–395. 10.1007/s40271-014-0065-0 25034612 10.1007/s40271-014-0065-0

[CR12] Byrne, D. (2022). A worked example of Braun and Clarke’s approach to reflexive thematic analysis. *Quality & Quantity,**56*(3), 1391–1412. 10.1007/s11135-021-01182-y

[CR13] Cerel, J., Brown, M. M., Maple, M., Singleton, M., Venne, J., Moore, M., & Flaherty, C. (2019). How many people are exposed to suicide? *Not Six. Suicide and Life-Threatening Behavior,**49*(2), 529–534. 10.1111/sltb.12450 29512876 10.1111/sltb.12450

[CR14] Cerel, J., McIntosh, J. L., Neimeyer, R. A., Maple, M., & Marshall, D. (2014). The continuum of “survivorship”: Definitional issues in the aftermath of suicide. *Suicide and Life-Threatening Behavior,**44*(6), 591–600. 10.1111/sltb.12093 24702241 10.1111/sltb.12093

[CR15] Crawford, M. J., Robotham, D., Thana, L., Patterson, S., Weaver, T., Barber, R., Wykes, T., & Rose, D. (2011). Selecting outcome measures in mental health: The views of service users. *Journal of Mental Health (Abingdon, England),**20*(4), 336–346. 10.3109/09638237.2011.577114 21770782 10.3109/09638237.2011.577114

[CR16] Davidson, P. M., Halcomb, E. J., & Gholizadeh, L. (2022). Introducing evidence-based practice in health care. In P. Liamputtong (Ed.), *Qualitative research methodology and evidence-based practice in public health* (4th ed., pp. 76–112). Oxford University Press.

[CR17] de Groot, M., & Kollen, B. J. (2013). Course of bereavement over 8–10 years in first degree relatives and spouses of people who committed suicide: Longitudinal community based cohort study. *BMJ (Online),**347*(7928), 13–13. 10.1136/bmj.f5519 10.1136/bmj.f5519 PMC3788666 24089424

[CR18] DeCuir-Gunby, J. T., & Schutz, P. A. (2017). Chapter 1 The role of theory in mixed methods research. In *Developing a mixed methods proposal: A practical guide for beginning researchers*. SAGE. 10.4135/9781483399980

[CR19] Department of Health. (2020). *Budget 2020–21 prioritising mental health—Enhancing suicide prevention*. Australian Government.

[CR20] Suicide Prevention Australia. (2023). Federal Budget fails those in crisis. https://www.suicidepreventionaust.org/federal-budget-fails-those-in-crisis/

[CR21] Greenhalgh, J., Gooding, K., Gibbons, E., Dalkin, S., Wright, J., Valderas, J., & Black, N. (2018). How do patient reported outcome measures (PROMs) support clinician-patient communication and patient care? A realist synthesis. *Journal of Patient-Reported Outcomes,**2*(1), 42–42. 10.1186/s41687-018-0061-6 30294712 10.1186/s41687-018-0061-6 PMC6153194

[CR22] Jackson, B., Wayland, S., Ball, S.-A., Lamperd, A., Potter, A., & Maple, M. (2024). Measuring the outcomes of support provided to people after a suicide or other sudden bereavement: A scoping review. *Death Studies*. 10.1080/07481187.2024.2419618 39509146 10.1080/07481187.2024.2419618

[CR23] Kaspersen, S. L., Kalseth, J., Stene-Larsen, K., & Reneflot, A. (2022). Use of health services and support resources by immediate family members bereaved by suicide: A scoping review. *Internation Journal of Environmental Research and Public Health*. 10.3390/ijerph191610016 10.3390/ijerph191610016 PMC9408753 36011651

[CR24] Kõlves, K., Zhao, Q., Ross, V., Hawgood, J., Spence, S. H., & de Leo, D. (2020). Suicide and sudden death bereavement in Australia: A longitudinal study of family members over 2 years after death. *Australian and New Zealand Journal of Psychiatry,**54*(1), 89–98. 10.1177/0004867419882490 31647307 10.1177/0004867419882490

[CR25] Maple, M., Cerel, J., Sanford, R., Pearce, T., & Jordan, J. (2017). Is exposure to suicide beyond kin associated with risk for suicidal behavior? A systematic review of the evidence. *Suicide & Life-Threatening Behavior,**47*(4), 461–474. 10.1111/sltb.12308 27786372 10.1111/sltb.12308

[CR26] Maple, M., Pearce, T., Sanford, R., Cerel, J., Castelli Dransart, D. A., & Andriessen, K. (2018). A systematic mapping of suicide bereavement and postvention research and a proposed strategic research agenda. *Crisis: THe Journal of Crisis Intervention and Suicide Prevention,**39*(4), 275–282. 10.1027/0227-5910/a000498 10.1027/0227-5910/a000498 29256269

[CR27] Ministry of Health. (2019). *Every Life Matters—He Tapu te Oranga o ia tangata: Suicide Prevention Strategy 2019–2029 and Suicide Prevention Action Plan 2019–2024 for Aotearoa New Zealand*. Wellington.

[CR28] Oster, C., Dawson, S., Kernot, J., & Lawn, S. (2023). Mental health outcome measures in the Australian context: What is the problem represented to be? *BMC Psychiatry,**23*(1), 24–24. 10.1186/s12888-022-04459-0 36627588 10.1186/s12888-022-04459-0 PMC9832818

[CR29] Pitman, A., Khrisna Putri, A., Kennedy, N., De Souza, T., King, M., & Osborn, D. (2016). Priorities for the development and evaluation of support after suicide bereavement in the UK: Results of a discussion group. *Bereavement Care,**35*(3), 09–116. 10.1080/02682621.2016.1254457

[CR30] Putri, A. K., Armstrong, G., Setiyawati, D., & Andriessen, K. (2024). Unveiling studies on self-healing practices for suicide loss survivors: A scoping review. *Death Studies*. 10.1080/07481187.2024.2304773 38259251 10.1080/07481187.2024.2304773

[CR31] Queensland Alliance for Mental Health. (2019). *Measuring outcomes in community mental health*. https://www.qamh.org.au/wp-content/uploads/MEASURING-OUTCOMES-IN-COMMUNITY-MENTAL-HEALTH-FINAL-VERSION.pdf

[CR32] Roe, D., Slade, M., & Jones, N. (2022). The utility of patient-reported outcome measures in mental health. *World Psychiatry,**21*(1), 56–57. 10.1002/wps.20924 35015343 10.1002/wps.20924 PMC8751576

[CR33] Schut, H., & Stroebe, M. (2011). Challenges in evaluating adult bereavement services. *Bereavement Care,**30*(1), 5–9. 10.1080/02682621.2011.555240

[CR34] Seivwright, A., Flatau, P., Adams, S., & Stokes, C. (2016). *The future of outcomes measurement in the community sector*. Bankwest Foundation.

[CR35] Trainor, L. R., & Bundon, A. (2021). Developing the craft: Reflexive accounts of doing reflexive thematic analysis. *Qualitative Research in Sport, Exercise and Health,**13*(5), 705–726. 10.1080/2159676X.2020.1840423

[CR36] Ussher, J. M., Hawkey, A. J., Perz, J., Liamputtong, P., Sekar, J. A., Marjadi, B., Schmied, V., Dune, T. M., & Brook, E. D. (2020). Crossing boundaries and fetishization: Experiences of sexual violence for trans women of color. *Journal of Interpersonal Violence,**37*, NP3552–NP3584. 32783523 10.1177/0886260520949149

[CR37] Weier, M., Wearring, A., & Kelly, M. (2022). *StandBy—Support after suicide social impact framework*. Centre for Social Impact UNSW.

[CR38] Williams, K., & Thompson, C. (2018). *Patient-reported outcome measures: Stakeholder Interviews*. ACSQHC.

[CR39] Wolpert, M. (2014). Uses and Abuses of Patient Reported Outcome Measures (PROMs): Potential Iatrogenic Impact of PROMs Implementation and How It Can Be Mitigated. *Administration and Policy in Mental Health and Mental Health Services Research,**41*(2), 141–145. 10.1007/s10488-013-0509-1 23867978 10.1007/s10488-013-0509-1 PMC3909250

