# Supplementary material for: Measuring What Outcomes Matters Most to People When Accessing Suicide Postvention Support: A Qualitative Study
Source: Community Ment Health J. 2025 Jan 21;61(6):1115–25. doi: 10.1007/s10597-025-01452-1 (PMC12228587; doi:10.1007/s10597-025-01452-1)
Supplement: Supplementary file 1 — Supplementary file1 (PDF 24 KB) [file 10597_2025_1452_MOESM1_ESM.pdf]

Online Resource 1.

Measuring What Outcomes Matters Most to People When Accessing Suicide Postvention Support: A Qualitative Study

Community Mental Health Journal

Bess Jackson*, Sarah Wayland, Shelley-Anne Ball, Myfanwy Maple

*Corresponding author:
University of New England, (School of Health), Armidale (NSW), Australia
bphilli8@myune.edu.au

Interview guide for focus groups.

Note: Script in italics; question in non-italics.

Type of question (question #)	**Group A** (staff)	**Group B** (LEAG)
Introductory (1)	For somebody accessing a service after a suicide loss, what is the most important support that the service can offer?	
Transition (2)	What changes have you observed in the lives of the people you support through the postvention service? How much can this be attributed to the support offered by the postvention service?	If you've accessed the postvention service support in the past, what changes did you observe in your life around that time? How much can this be attributed to the support offered by the postvention service?
Transition (3)	Can you think about a time that you felt the postvention service was not able to provide the support that somebody needed?<br><br>*If you can, please tell me about it.*	If you've accessed the postvention service support in the past, was there ever a time that you felt the postvention service was not able to provide the support that you needed?<br><br>*If you can, please tell me about it.*
Focus questions (4)	*Recent research on the postvention service suggests that the program is more impactful for people who have lost their person less than 12 months ago.*<br><br>How do support needs differ after the first 12 months?	
Focus questions (5)	*Your answers to the last few questions are useful for the postvention service to know ongoing - what's important, what changed, and where the gaps were.*<br><br>*It's not practical for the postvention service to hold focus groups like I'm doing today, so I am wanting to devise another way for the postvention service to understand and measure outcomes.*<br><br>How do you think the postvention service measure outcomes? Be as specific as possible (e.g., timing, frequency, modality, type of questions).	
Focus questions (6)	*Data collected from outcome measures can serve various purposes, such as guiding individual support, assessing program effectiveness, or understanding the impacts the postvention service has on the community. Each purpose may require a different approach. For instance, a tool suitable for assessing program effectiveness might not fully address the needs of therapeutic support.*<br><br>*Considering these different applications, we'd like to hear your perspective*.<br><br>Which purpose should be prioritised when designing and selecting outcome measurement tools? Do you think there's a balance that needs to be struck between these purposes?	

Online Resource 1.

Summarising question (7)	Think back to the ideas that we've discussed today. What concerns would you have around implementing any of these? How can these concerns be alleviated?	Think back to the ideas that we've discussed today. What concerns would you have around completing or participating in any of these? How can these be alleviated?
Closing question (8)	Is there anything else we should have talked about today but didn't? Please feel free to share these thoughts.	
